# Managerial Challenges in Digital Health: Bibliometric and Network Analysis

**DOI:** 10.2196/57980

**Published:** 2025-06-19

**Authors:** Quentin Garçon, Benjamin Cabanes, Cédric Denis-Rémis

**Affiliations:** 1 Institut des Hautes Etudes pour l’Innovation et l’Entrepreneuriat Mines Paris PSL University Paris France; 2 i3-CRG Ecole Polytechnique Institut Polytechnique de Paris Palaiseau France

**Keywords:** bibliometrics, digital health, mobile health, mHealth, telemedicine, management, adoption, ecosystems, privacy, literature review, ethics, health challenges

## Abstract

**Background:**

Digital health has emerged as a transformative force in modern health care systems; these systems have witnessed a surge in technological innovations and solutions over the past 2 to 3 decades. Some studies have provided overviews of keywords and journals that shed light on the current state of digital health research, and there is an increasing focus on this field of study, even in the literature on business, management, and managerial challenges. Papers and reviews are needed on challenges in digital health related to organization, management, and adoption of technological innovations, as there are currently no formal analyses or structured reviews.

**Objective:**

Given the existing focus of the business and management literature on digital health, there is a need to unravel managerial challenges in digital health. By highlighting foundational themes and challenges in management science for digital health, our objective is to contribute nuanced insights into influential studies and the structure of knowledge in this interdisciplinary domain.

**Methods:**

To delineate the evolving landscape of digital health management and highlight the main challenges, we conducted a comprehensive bibliometric analysis. Our analysis considered peer-reviewed, English-language papers or reviews in the management field that focused on digital health or closely related concepts. To better understand the dataset, we conducted a performance analysis. Then, we created a co-citation network using BibExcel and analyzed it by clustering the papers using the Louvain algorithm in Gephi.

**Results:**

Of 1186 papers about digital health or closely related concepts published between 1994 and 2022, 520 (43.8%) were included in the co-citation network and 468 (39.5%) were placed in 4 significant clusters (>1% of the total number of nodes). The mere existence of the clusters shows that different managerial challenges have distinct research communities. The 4 clusters were (1) user adoption and engagement in mHealth, (2) adoption and trust in mHealth services, (3) digital transformation in health care, and (4) implementation challenges and ethical considerations. There are interdependent managerial challenges in digital health, and a dynamic literature review provides a more precise understanding of what is at stake (eg, adoption studies) and upcoming challenges (eg, ethical considerations).

**Conclusions:**

Our co-citation analysis unveiled evolving themes in the literature on digital health management. The exploration of clusters suggested dynamic shifts related to ethical considerations, health care organizations, and societal acceptance. We encourage further research on these topics and exploration of the intricate facets of the literature on digital health management. We hope that this study provides a more comprehensive understanding of the literature and will provide researchers insights into the principal challenges and unidentified gaps, such as the novel cluster on ethics, or the need for intercluster research to create links between research communities.

## Introduction

### Background

According to the US Food and Drug Administration, digital health includes categories such as mobile health (mHealth), health IT (HIT), wearable devices, telehealth and telemedicine, and personalized medicine [1]. Digital health can be explained as “the proper use of technology for improving the health and wellbeing of people at individual and population levels, as well as enhancing the care of patients through intelligent processing of clinical and genetic data” [2]. The evolution of medical paradigms such as preventive medicine for aging populations, digital health, and eHealth has led to inspiring shifts and revolutions for both researchers and governments and has created various new challenges for management researchers [3,4]. Key managerial challenges in digital health include empowering patients, enhancing health literacy, improving access to digital solutions in underserved areas, and enabling better patient monitoring, all of which require innovative organizational approaches to optimize health care outcomes.

### Prior Work

Worldwide, an expanding number of researchers are actively engaging in the exploration, application, evaluation, and leveraging of the advantages associated with digital health and its diverse technological components in investigations concerning individuals, populations, and health care organizations [5]. This heightened engagement is discernible through the prevalent incorporation of the term “digital health” as a keyword in published, peer-reviewed literature [6]. Over the past 3 decades, there has been a noticeable surge in both the quantity and diversity of research initiatives, study protocols, published works, and specialized journals, all playing pivotal roles in the realm of digital health. Based on the growth in the use of this keyword, various features have been identified as part of “digital health,” including the 10 *E*’s: efficiency, enhancing quality, evidence based, empowerment, encouragement, education, enabling, extending, ethics, and equity [7]. Since the study of digital health can be broad, encompassing the entire field of medicine, or specific, targeting interventions like mobile health apps [8], management researchers also study the organization of digital health and its consequences for the health sector, as well as the ethical point of view [9]. Many academic papers in the management field engage in describing and unravelling shifts since COVID-19 [10-12] or the global changes allowed by digital health [13]. However, this kind of research remains very niche in management or organizational studies, and there is no real description of the principal managerial challenges in digital health.

### Goal of This Study

The digital health literature has offered several reviews and review types in recent years, particularly on topics such as use of digital health, technology acceptance, and mobile apps [4,14]. However, research on digital health management remains limited and is often project driven [15,16]. While such studies provide valuable insights into specific management issues in digital health, they are highly focused. Despite the abundance of studies, none have systematically explored knowledge generation for management issues in digital health to identify and address its key managerial challenges. This analysis aims to formalize the field’s knowledge structure and identify key challenges and thematic areas in digital health management research, supporting scholars and managers in addressing critical issues and engaging with relevant research communities. The scope is broad because it includes all potential research in digital health management, and the focus is on the present main challenges in research. Thus, a performance analysis followed by a co-citation network analysis seemed to be the best method to answer the question [17]. A co-citation network was also used to delineate clusters and identify key challenges.

By conducting this analysis of business and management papers about digital health, we answer the following question: What are the main managerial challenges in digital health?

## Methods

### Research Design

To better understand the foundational themes in a research field and its global structure, it is recommended to use a co-citation analysis. This method allows researchers to delineate thematic clusters, leaving niche publications out of the themes [17]. To perform a co-citation analysis, a dataset of papers and their references has to be collected. Since the completeness of Google Scholar as a complete source for clinicians is debated, and it is not suited for citations, co-citations, or author analysis [18,19], it was not used for this analysis. Even though PubMed is widely used by clinicians, it suffers from the same disadvantage of not being well suited for this type of analysis. Web of Science and Scopus both offer a wide range of journal and visualization tools, including inclusion and exclusion criteria. However, Scopus has substantially better coverage, especially in the field of social sciences, which encompasses management science [20]. For this reason, only Scopus was used in this study.

The strategy to select data was to find the most-used keywords using different requests and selecting only digital health–related keywords. After using these keywords as the final search string (Textbox 1), the results were exported and studied using performance analysis. The date were then organized as a co-citation network using BibExcel (Universität Wien) and visualized with Gephi (NetBeans).

**Scopus searches.**

**Search 1—Same Scopus request adding the publication stage (published), the type of publication (papers or reviews), and the language (English)**

TITLE-ABS-KEY (“digital health”) AND (PUBYEAR <2023) AND (LIMIT-TO (DOCTYPE, “ar”) OR LIMIT-TO (DOCTYPE, “re”)) AND (LIMIT-TO (PUBSTAGE, “final”)) AND (LIMIT-TO (LANGUAGE, “English”))

**Search 2—Example of equivalent Scopus request with “ehealth” instead of “digital health”**

TITLE-ABS-KEY (“ehealth”) AND (PUBYEAR <2023) AND (LIMIT-TO (DOCTYPE, “ar”) OR LIMIT-TO (DOCTYPE, “re”)) AND (LIMIT-TO (PUBSTAGE, “final”)) AND (LIMIT-TO (LANGUAGE, “English”))

**Search 3—Final Scopus request**

(KEY (”digital health”) OR KEY (ehealth) OR KEY (telemedicine) OR KEY (telehealth) OR KEY (mhealth) OR KEY (”Mobile Health”) OR KEY (”Electronic Health Record”) OR KEY (”Medical Technology”) OR KEY (”Medical Informatics”) OR KEY (”Electronic Health Records”) OR KEY (telemonitoring) OR KEY (teleconsultation) OR KEY (”Digital Health Intervention”)) AND (LIMIT-TO (SUBJAREA, “BUSI”)) AND (PUBYEAR <2023) AND (LIMIT-TO (PUBSTAGE, “final”)) AND (LIMIT-TO (DOCTYPE, “ar”) OR LIMIT-TO (DOCTYPE, “re”)) AND (LIMIT-TO (LANGUAGE, “English”))

### Data Collection

For data collection, all peer-reviewed journals were considered. The first search used “digital health” as a keyword in the title or abstract. Since entries were still being added to the 2023 database until approximately March or April 2024, we excluded entries from 2023 or later for more consistency and reproducibility. We also excluded papers that were not in English. For more consistency, we only selected papers or reviews that were published (Textbox 1, search 1).

For comparison and keyword selection purposes, other requests were tried with the same criteria but with “ehealth” as a keyword in the title or abstract (Textbox 1, search 2).

Among the papers returned by the search, we conducted a basic keyword analysis: we looked at the most-used keywords in all the papers [6] and looked for keywords that were related to digital health. We selected the keywords that were common in the keyword lists from both the digital health search (Textbox 1, search 1) and the eHealth search (Textbox 1, search 2) to select our final keywords (Multimedia Appendix 1).

After this analysis, we were left with these keywords: *digital health*, *ehealth*, *telemedicine*, *telehealth*, *mhealth*, *medical technology*, *medical informatics*, *telemonitoring*, *teleconsultation*, and *digital health intervention*. A new search was then performed to obtain all papers using these words, but the search was limited to the keywords to make sure that the search terms were not just mentioned in the abstracts of papers not related to digital health. For readability and consistency purposes, we kept the same criteria (English-language papers or reviews published before 2023). Since the object of this study was to analyze the main managerial challenges and potential thematic clusters in digital health management research, we only included papers in the field of business, management, and accounting to perform our final search (Textbox 1, search 3).

At this point, a detailed manual review confirmed the absence of duplicates due to the use of a Boolean request in Scopus, which inherently prevents duplication. To ensure that all papers were relevant and suitable for the performance analysis, a full title screening of the 1186 returned papers was conducted. No papers were excluded, as all were aligned with the research scope. Since the goal of the study was to understand the fundamental knowledge structure of managerial research in digital health, retaining a broad dataset was essential for robust performance and co-citation analysis.

In summary, only Scopus was used as the source of papers. Only reviews or papers were included, and conference communications or duplicates of previous versions were excluded. Since the searches and exporting of the results took place during 2023, papers from 2023 and afterward were excluded to obtain complete years for statistical purposes. Only English-language papers in the category of business, management, and accounting were included.

The metadata of the 1186 papers were exported from Scopus to Microsoft Excel for the bibliometric analysis and to BibExcel for network generation.

### Data Analysis

After extracting the data in RIS, CSV, and text formats from Scopus, we conducted the first analysis: the performance analysis.

The data were not yet shaped to conduct a network or co-citation analysis. In Microsoft Excel, other fields of data (eg, year, country, authors, citation count, and source) were added to the table to allow for further analysis based on sorting, filtering, keyword analysis, and basic graph generation. BibExcel was then used to create co-citation networks and extract the links and nodes to be visualized [21].

Thereafter, all data were imported to Gephi to be visualized as a network. The created co-citation graph was not oriented. The ForceAtlas2 algorithm was used to obtain better visualization. A random-color scale was generated to identify clusters, and node sizes were assigned according to their degree centrality. No further treatment was added for visualization.

We used Gephi to calculate both the degree centrality and the modularity classes. “Degree centrality” measures the number of links for each node, “closeness centrality” measures the communication efficacy in a network, “betweenness centrality” measures the number of times a node is in the shortest path, “closeness centrality” measures the efficacy with which a node communicates with other nodes in a specific area, and Katz or eigenvector centrality measures the indirect influence of neighbors on each node. Given the aim of the study, we decided to use degree centrality rather the other types of centrality based on its ability to characterize the number of shared citations. Even if the co-citation PageRank algorithm can be used in co-citation analysis [22], the most relevant value to quantify the representativeness of a node inside a cluster is degree centrality.

### Statistical Analysis

#### Power

To identify clusters, we used the Louvain algorithm from Gephi based on a cluster’s modularity class [23]. This algorithm is built on the idea of searching for a high degree of local density inside a network [24].

Citation analysis evaluates the number of citations received by papers and is commonly used to rank journals and researchers, reflecting their impact on scientific research [25]. Although it has faced criticism [26], it remains a widely used method for literature analysis, particularly for identifying influential authors, journals, or papers within specific research domains. In this study, citation counts were not used to assign names to the clusters but rather to identify influential papers within each cluster, thereby highlighting the main managerial challenges.

For network analysis, degree centrality was used instead of the total number of citations for intracluster classification of papers. Intracluster ranking of degree centrality is used to find papers that are representative of the cluster. Extracluster representations are used to show the relative density of each cluster.

#### Data Visualization

For data visualization, after having processed all papers (n=1186) to create the co-citation network, we were left with 520 papers (nodes) and 2667 links. Some of the papers had no co-citation links with each other, meaning that they did not share any bibliography. Papers with no links to other papers (isolated nodes) were removed along with nodes that were not in clusters, representing at least 1% (12/1186) of the total number of nodes. The results of the network analysis are thus based on the 468 remaining papers (Multimedia Appendix 2). No more node exclusion occurred after this step.

## Results

### Performance Analysis

**Figure 1:**
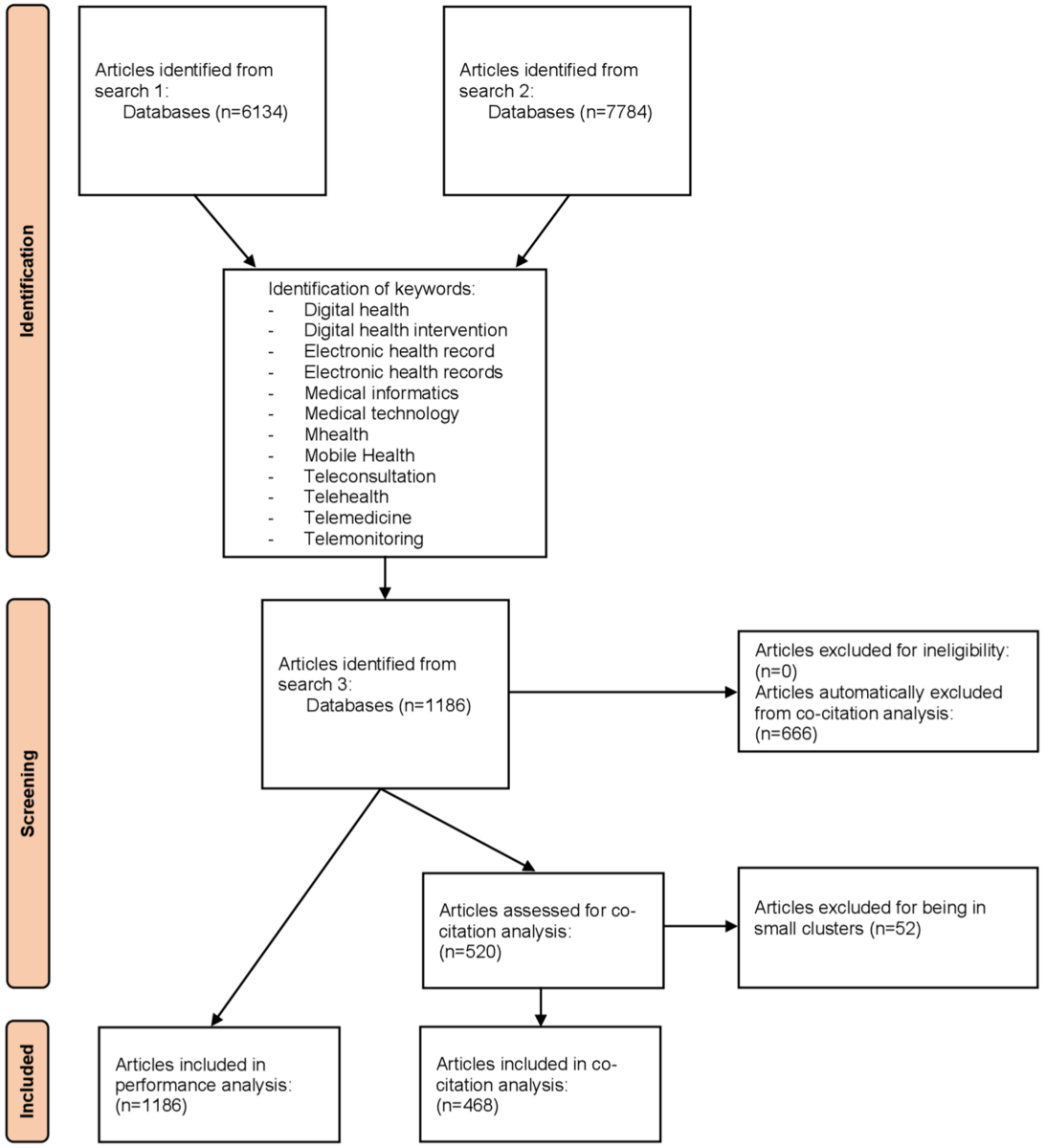
Methodology description from data collection to data analysis.

**Figure 2:**
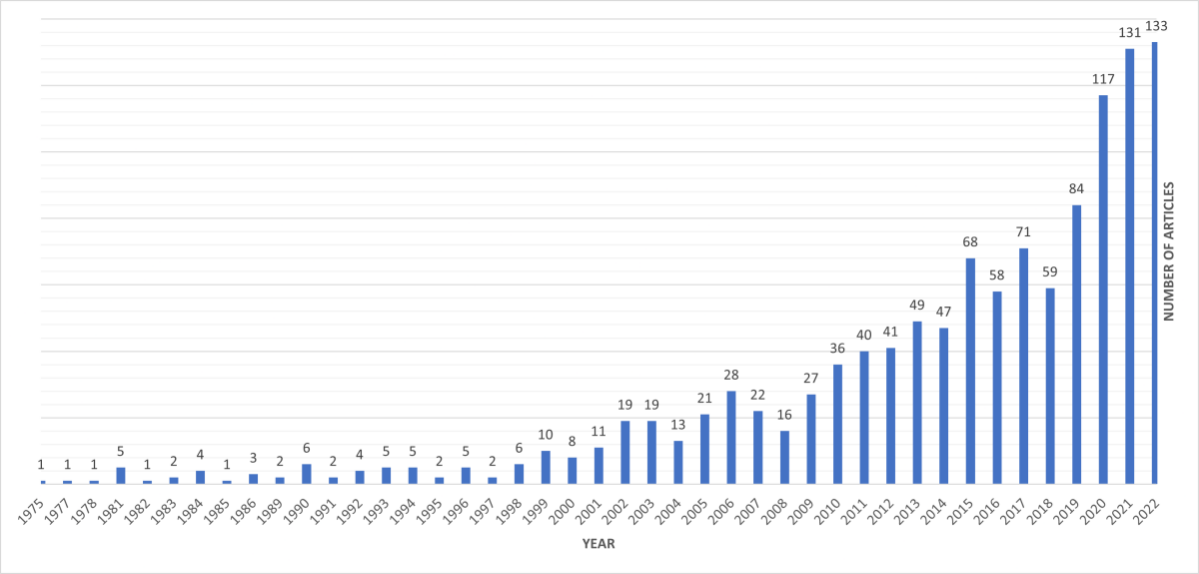
Year of publication of the 1186 papers in the bibliometric analysis.

For the period up to 2022, the final search (Textbox 1, search 3) returned 1186 English-language papers and reviews that were all final published versions (Figure 1). The oldest paper was one from 1975 and the most recent were 133 from 2022 (Figure 2). The substantial increase in publications on digital health management after 2010 might reflect the growing prioritization of digital technologies in health care systems worldwide. This trend aligns with key initiatives such as the HITECH Act in the United States, which incentivized the adoption of electronic health records [27], and the European Union’s Digital Agenda for Europe, which promoted eHealth as part of health care modernization [28]. The World Health Organization’s Global Observatory for eHealth, created in 2005 [29], further emphasized the importance of digital tools in addressing global health care challenges and started updating an online directory of eHealth-related national policies in 2010 [30]. These policies and initiatives likely contributed to the surge in research and publications in this field.

All 1186 papers were published in the subject area of business, management, and accounting. A total of 25 other subject areas were found, with the highest being medicine, with 364 papers, followed by computer science, with 311 papers (Table 1; Multimedia Appendix 3). Other subject areas that were highly represented (ie, comprising more than 10% of the papers) included social sciences (n=268 papers), decision sciences (n=248 papers), nursing (n=158 papers), engineering (n=145 papers), and economics, econometrics, and finance (n=128 papers).

**Table 1:** Top secondary subject areas of the 1186 papers included in the bibliometric analysis.

Secondary subject area	Papers, n (%)
Medicine	364 (30.7)
Computer science	311 (26.2)
Social sciences	268 (22.6)
Decision sciences	248 (20.9)
Nursing	158 (13.3)
Engineering	145 (12.2)
Economics, econometrics, and finance	128 (10.8)
Psychology	92 (7.8)

Among these papers, 101 were considered reviews, meaning that the research was not based on primary data but on secondary published data. More than 160 different sources published these papers, from each source publishing from 1 to 54 papers (Table 2; Multimedia Appendix 4). The top 3 most represented sources were *Health Care Management Review* (n=54 papers), the *Journal of Healthcare Management* (n=54 papers), and the *Journal of Commercial Biotechnology* (n=45 papers).

**Table 2:** Top sources of the 1186 papers included in the bibliometric analysis.

Source journal	Papers, n (%)
*Health Care Management Review*	54 (4.6)
*Journal of Healthcare Management*	54 (4.6)
*Journal of Commercial Biotechnology*	45 (3.8)
*Technological Forecasting and Social Change*	38 (3.2)
*International Journal of Health Care Quality Assurance*	32 (2.7)
*Contract Pharma*	25 (2.1)
*Science and Engineering Ethics*	25 (2.1)
*Technology in Society*	23 (1.9)
*International Journal of Information Management*	21 (1.8)
*Decision Support Systems*	19 (1.6)

An analysis of keywords with no cluster-focus approach would have been biased, since the request was keyword based. Hundreds of keywords appeared; the top 5 most represented were *human*, *paper*, *humans*, *medical technology*, and *telemedicine* (Multimedia Appendix 5). For this reason, a cluster-focus keyword analysis was conducted. The keywords of research papers are human generated, so one cannot expect them to be homogeneous among research teams, universities, laboratories, and, a fortiori, countries. We conducted a grammatical, semantic, and conceptual analysis to standardize terminology and consolidate related terms into cohesive categories. This involved identifying variations in spelling, language, and word usage across different contexts and subsequently grouping synonymous terms and phrases to ensure consistency and accuracy in our analysis (Multimedia Appendix 6).

Each research paper was affiliated with the countries of its authors, meaning that a single paper had multiple country affiliations if it had authors from different countries or international partnerships. To be precise, if a paper had 1 author, then the author affiliation was the paper affiliation; if the paper had 2 or more authors from the same country, then the affiliation of the paper was this country and was counted once; otherwise, if the paper had 2 or more authors from different countries, then the paper was assigned each different country as an affiliation. In total, 77 countries were represented (Multimedia Appendix 7). We categorized these countries over 6 geographical zones: North America, South America, Europe, Asia, Oceania, and Africa (Table 3). The top 5 countries were the United States (n=492 papers), United Kingdom (n=85 papers), China (n=79 papers), Australia (n=63 papers), and India (n=59 papers). There were 112 papers that were not automatically affiliated to a country, so they were counted as “undefined.” All papers were written in English, although 2 papers were written either in Spanish or Portuguese as well as English. Oceania, Africa, and South America were significantly underrepresented, with only 10.1% of affiliations.

**Table 3:** Affiliation zones of the 1186 papers included in the bibliometric analysis.

Geographical zone	Papers, n (%)
North America	546 (46.0)
Europe	354 (29.8)
Asia	341 (28.8)
Oceania	69 (5.8)
Africa	32 (2.7)
South America	19 (1.6)

### Co-Citation Analysis

**Figure 3:**
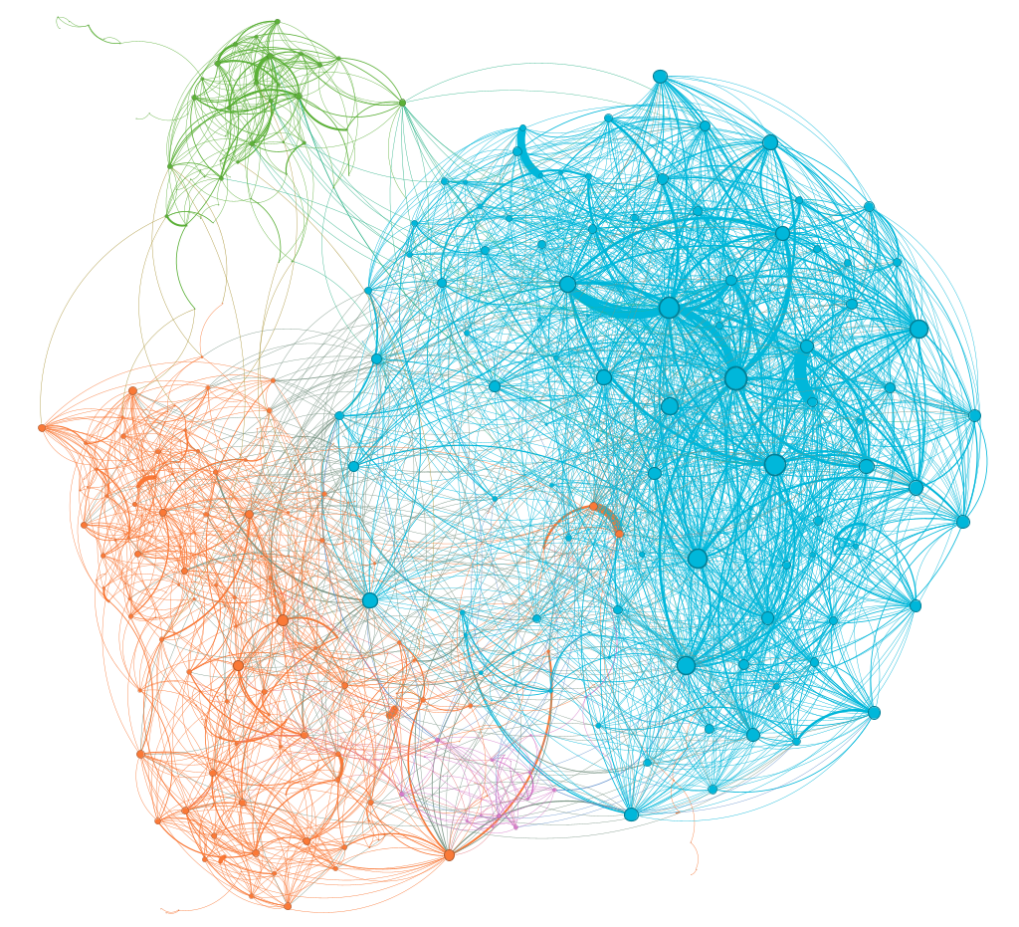
Cluster representation of the 468 papers using Gephi. A description of the clusters is as follows: modularity class 1 (light blue, top right) had 162 nodes (34.6%), modularity class 2 (green, top left) had 57 nodes (12.2%), modularity class 3 (orange, bottom left) had 223 nodes (47.6%), and modularity class 4 (purple, bottom center) had 26 nodes (5.6%).

Using these 1186 papers, a co-citation analysis was conducted with BibExcel to identify potential clusters. Only 520 of the 1186 papers were represented as nodes on the network because of the existence of co-citation links. The Louvain algorithm in Gephi identified multiple proposed clusters. The weights used to compute the clusters were those from the co-citation analysis. After the identification of clusters and the removal of clusters having less than 1% of the number of nodes (ie, less than 5 nodes), 468 papers were left in the analysis. After applying this exclusion criterion, 4 clusters were left (Figure 3). It is important to note that all top-cited papers and those with the highest degree centrality were from the 4 clusters comprising 468 papers, with none found in the excluded set of 52 papers. Since nodes were removed, the degree centrality was updated. For readability, every degree centrality was then based on the 468 papers in the 4 clusters and not the 520 papers. At this point, we performed a full abstract screening to have a better understanding of the diversity of the clusters and to ensure that all papers were relevant.

**Figure 4:**
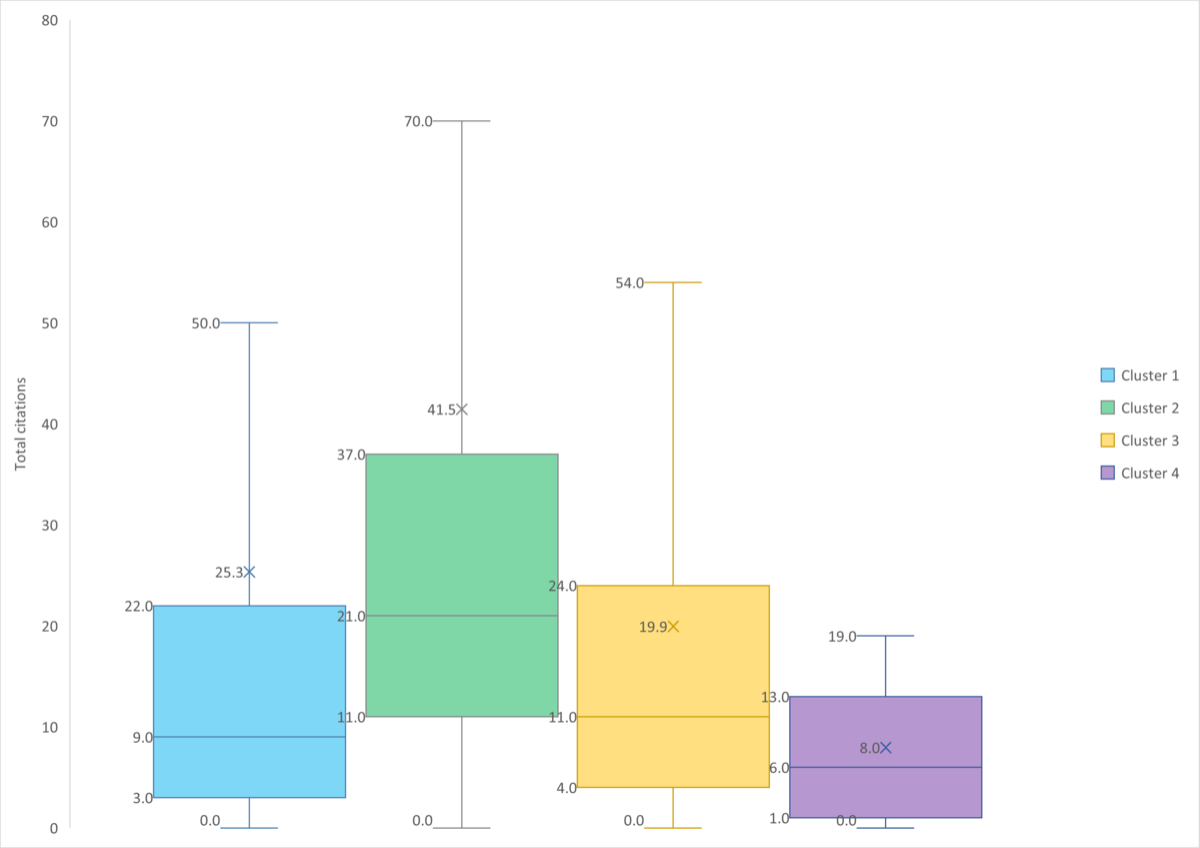
Box plot of the total citation distribution for each cluster showing position indicators: medians (horizontal lines), averages (crosses), IQRs (boxes) and extended IQRs (whiskers) for both total citations and degree centrality.

**Figure 5:**
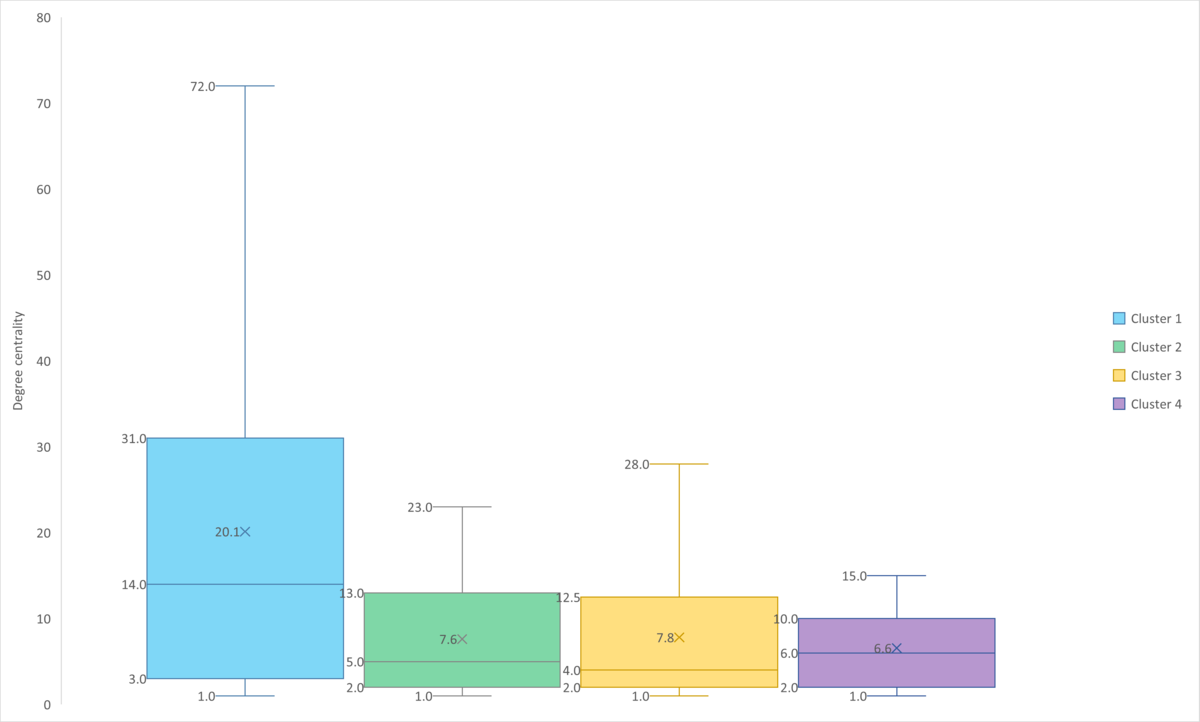
Box plot of the global degree centrality distribution for each cluster showing position indicators: medians (horizontal lines), averages (crosses), IQRs (boxes) and extended IQRs (whiskers) for both total citations and degree centrality.

To better understand the diversity of papers in terms of citation rank and degree centrality, we also represented these data as box plots (Figures 4 and 5) showing position indicators such as the median, average, IQR, and extended IQR (EIQR) for both total citations and degree centrality. In this study, the IQR represents the spread from the first quartile (Q1) to the third quartile (Q3). To account for potential outliers, we defined EIQR as the range from Q1−1.5×IQR to Q3+1.5×IQR, following the Tukey fences method. The minimum value for the EIQR cannot be less than the minimum value of each cluster. For the total citations, this value is 0; for degree centrality, this value is 1 (since 0 would be a node not in any cluster). Minimum and maximum values for both total citations and degree centrality are given in Table 4.

**Table 4:** Position and dispersion indicators of total citations and degree centrality for each cluster.

Modularity class		Average	Minimum	Maximum	Median (IQR)	Extended IQR
**Total citations**						
	1	47.7	0	1297	11.0 (4.0-30.5)	0-69.0
	2	41.9	0	353	24.0 (12.0-38.0)	0-76.0
	3	32.3	0	650	14.0 (5.0-30.0)	0-67.0
	4	9.8	0	58	6.0 (1.0-13.8)	0-32.0
**Degree centrality**						
	1	18.9	1	78	13.5 (2.0-29.3)	1-67.0
	2	7.4	1	23	5.0 (2.0-13.0)	1-23.0
	3	7.6	1	38	4.0 (2.0-11.0)	1-24.0
	4	6.0	1	15	5.0 (2.0-8.5)	1-15.0

The mere presence of clusters constitutes a significant finding, as scientific fields can exhibit varying degrees of cohesion, ranging from uniform interconnections among papers to complete absence of clustering.

The same method was consistently applied across all clusters to assign each a name or title based on the papers they contained and to identify the main challenges addressed by the most significant papers within each cluster. To determine a cluster name, the papers were ranked according to their degree centrality, emphasizing those most significant for delineating the cluster. The abstracts of all papers were reviewed, followed by a detailed reading of the 10 papers with the highest degree centrality. To identify the main challenges, the papers were ranked by their total number of citations, and the 10 most-cited papers of the decade within each cluster were read in detail to extract the key challenges.

### Cluster 1: User Adoption and Engagement in mHealth

The first cluster, represented in light blue in Figure 3, had 162 nodes (34.6% of the total number of nodes) which made it the second-biggest cluster. With a median total citations of 11, more than 50% of the papers were less cited than the 75% most-cited papers in cluster 2. Almost 75% of the papers from cluster 4 were also less cited than 50% of the most-cited papers from cluster 1. For several highly cited papers, the average total citations was 47.7, with an overall high dispersion. The dispersion was quite similar to cluster 3.

In terms of degree centrality, with an average of 18.9, this was by far the most highly connected cluster among its own nodes. It contained globally higher degree centrality, with a maximum degree centrality at 78 nodes, but it was also the most dispersed. The median degree centrality was 13.5, higher than Q3 in the other clusters, and 50% of the most connected papers were more connected than 75% of the papers in the other clusters.

Through content analysis, we were able to label cluster 1 as “user adoption and engagement in mHealth.” Cluster 1 also examined the adoption of mHealth services in low-income countries, specifically India and Bangladesh. Cluster 1 explored emotional bonding with mHealth apps, gamification, and cross-country analysis of adoption patterns. Additionally, it investigated the impact of these technologies on the quality of life, preventive health care, and patient knowledge creation through telemedicine technologies. Cluster 1 included many papers that used the technology acceptance model (TAM), along with papers addressing information privacy (Table 5).

**Table 5:** Top 3 papers from cluster 1 according to their degree centrality.

Degree centrality	Title	Authors, Year	Total citations^a^	Principal findings
78	“Do mobile health (mHealth) services ensure the quality of health life? An integrated approach from a low-income country context”	Alam et al [31], 2022	17	The principal factors having a significant impact on adoption of mHealth services among generation Y are performance expectancy, effort expectancy, facilitating conditions, social influence, hedonic motivation, system quality, and service quality. Consequently, it is important to build trust and satisfaction to improve the health-related quality of life.
72	“Adoption of mobile fitness and dietary apps in India: An empirical study”	Sampat et al [32], 2020	5	The most important factors for mobile fitness and dietary app adoption are perceived usefulness and trust.
71	“Factors influencing the adoption of mHealth services in a low-income country: A patient-centric study”	Alam et al [33], 2020	189	The behavioral intention to adopt mHealth services in a low-income country is positively influenced by performance expectancy, social influence, facilitating conditions, and perceived reliability. Other factors, such as effort expectancy and price value, have no significant influence on behavioral intention to adopt mHealth services. Gender is also discussed as having a moderating effect on mHealth service adoption.

^a^Last updated on February 26, 2024.

This content analysis was strengthened by an independent keyword analysis (Table 6). Apart from *mhealth*, *ehealth* and *telemedicine*, which were included in the inclusion criteria, various keywords appeared in the top 10, including keywords related to adoption, such as *adoption*, *adoption model*, *adoption fit*, and *adoption intention*; other keywords were related to the unified theory of acceptance and use of technology (UTAUT), including *utaut*, *utaut2*, and *utaut model*, and behavior, including *behaviour*, *behavior*, *behavioral*, *behavior intention*, and *theory of planned behavior*.

**Table 6:** Top 10 grouped keywords in cluster 1.

Grouped keywords	Count, n
mhealth	64
adoption	50
ehealth	40
telemedicine	31
ehr	20
utaut	19
behaviour	14
hit	12
research	11
literacy	11

Taking into account the most-cited papers from this decade, we can state that adoption, and especially mHealth adoption, is one of the most important challenges. Using gamification to allow patients to benefit more from a technology seems to be a solution, while both the utilitarian aspect and hedonic aspect seem to have significant roles, with slight differences [34]. Using the TAM methodology, various studies have shown that user adoption is influenced by many factors. In particular, perceived usefulness, perceived ease of use, perceived vulnerability, and perceived severity had a significant influence on patients’ attitudes, while perceived usefulness, perceived ease of use, subjective norms, trust, perceived risk, and attitude had more influence on behavioral intention [35]. However, even though these factors are known, optimizing each of them, especially trust in high-income countries and literacy in low-income countries, remains an important managerial challenge to help patients adopt these new health technologies, improve patient empowerment, and reduce social inequalities [36,37].

### Cluster 2: Adoption and Trust in mHealth Services

The second cluster, represented in green (Figure 3), had 57 nodes (12.2% of the total number of nodes) which makes it the third-largest cluster. The median total citations of 24 was the highest among all clusters, but it also had the highest dispersion; more than 75% of the papers were more cited than 75% of papers in cluster 4, and they were also more cited than 50% of the papers in cluster 1.

In terms of degree centrality, with an average of 7.4 and a median of 5.0, the cluster was well connected but did not contain the highest-degree papers. More than 75% of these papers had a lesser degree than 50% of the papers in cluster 1.

Through content analysis, we were able to label cluster 2 as “adoption and trust in mHealth services,” which seems quite similar to cluster 1. However, cluster 2 explored factors influencing mHealth adoption, including emotional attachment, trust, and the paradox between privacy and personalization. Cluster 2 was related to the acceptance of telemedicine services in different cultural contexts and incorporated artificial intelligence in health care, examination of user engagement, and learning perspectives within mHealth apps. Numerous research papers from cluster 2 were based on the UTAUT or addressed the risk-trust relationship to predict the intention to use mobile health apps and sensors in the medical context (Table 7).

**Table 7:** Top 3 papers according to their degree centrality from cluster 2.

Degree centrality	Title	Authors Year	Total citations^a^	Principal findings
23	Effects of emotional attachment on mobile health-monitoring service usage: an affect transfer perspective	Xiaofei et al [38], 2021	42	Patients with chronic illnesses develop emotional attachments to mobile health-monitoring services. Their satisfaction with the services influences their affective evaluation of using the services. The effects of device satisfaction and feedback satisfaction on the services’ perceived value is positively moderated by the patients’ health rationality.
21	An extension of technology acceptance model for mHealth user adoption	Rajak and Shaw [39], 2021	38	Patient trust in mHealth services positively impacts perceived usefulness and perceived ease of use. Perceived risk, resistance to change, and perceived physical condition negatively impact behavioral intention. Social influence, trust, and behavioral intention positively influence adoption of mHealth services.
19	The role of trust in intention to use the IoT in eHealth: application of the modified UTAUT in a consumer context	Arfi et al [40], 2021	122	The risk-trust relationship is the principal factor in Internet of Things adoption for eHealth, whereas performance expectancy has no impact on intention to use the Internet of Things for eHealth.

^a^Last updated on February 26, 2024.

Again, the content analysis was strengthened by the independent keyword analysis (Table 8). Apart from *mhealth*, *ehealth*, *EHR* (electronic medical records) and *telemedicine*, which were part of the inclusion criteria, keywords that appeared in the top 10 included *adoption* (including *adoption*, *adoption model*, *adoption fit*, *adoption intention*…), *behaviour* (including *behaviour*, *behavior*, *behavioral*, *behavior intention*, *theory of planned behaviour*…), *UTAUT* (including *utaut*, *utaut2* and *utaut model*), *apps* (including *apps*, *mobile apps* and *health apps*), and *trust* (including *trusting* and *virtual trust*).

**Table 8:** Top 11 grouped keywords in cluster 2.

Grouped keywords	Count
mhealth	18
ehr	11
adoption	9
healthcare	7
telemedicine	7
ehealth	7
behaviour	6
hit	6
apps	5
covid	4
management	4

Taking into account the most cited papers from this decade, we can state that adoption is again a main focus for academics but with a different angle. With many fewer papers on mHealth, other fields became prominent, such as AI, telemedicine, and EHRs, with privacy and trust also being studied. While performance expectancy seems to have had no significant impact on the intention to use digital health objects (ie, the Internet of Things), trust has become a significant determinant of adoption, and this has become even more true in younger populations [40,41]. Also, more than demographics, social or urban situations impact the way patients perceive and use digital health technology such as telemedicine. In particular, in low-income countries, even though privacy was found to be an insignificant determinant of use intention, parameters such as perceived usefulness, perceived ease of use, social influence, facilitating conditions, and trust were significant determinants for the acceptance of telemedicine services [42]. This information should lead to adaption and personalization of the user experience with consideration of various factors, which could be a challenging mission for policy makers.

### Cluster 3: Digital Transformation in Health Care

The third cluster, represented in orange in Figure 3, had 223 nodes (47.6% of the total number of nodes) which made it the biggest cluster. With a median total citations of 14, more than 50% of the papers were more cited than 75% of the papers in cluster 4. The maximum number of total citations was the second highest among all clusters, with an overall high dispersion.

In terms of degree centrality, with a median of 4.0 and a maximum degree of 38, this cluster had some highly connected nodes but also some very poorly connected nodes. Again, more than 75% of these papers had a lesser degree than 50% of the papers from cluster 1.

Through content analysis, we were able to label cluster 3 as “digital transformation in health care.” Cluster 3 revolves around the digital transformation of the health care industry. It explores various aspects of technology adoption, including patient engagement through telemedicine, EHR assimilation, and the impact of HIT on health care quality and cost. Cluster 3 was related to ethical considerations in digital health care, focusing on responsible design, patient empowerment through digital health trackers, and, most importantly, the challenges of introducing digital technologies into health care ecosystems. The research papers were mostly on value co-creation, ecosystems, and implementing HIT in organizations (Table 9).

**Table 9:** Top 3 papers according to their degree centrality from cluster 3.

Degree centrality	Title	Authors Year	Total citations^a^	Principal findings
38	The digital transformation of the healthcare industry: exploring the rise of emerging platform ecosystems and their influence on the role of patients	Hermes et al [43], 2020	111	New roles are discovered in the digital transformation of the health care industry. The role of patients as cocreators of value is evolving, and this new role in the context of digitalization of health care is related to tackling simple linear value chains in a platform-mediated multisided market.
37	Value cocreation in service ecosystems: investigating health care at the micro, meso, and macro levels	Beirão et al [44], 2017	191	Resource access, resource sharing, resource recombination, resource monitoring, and governance or institution generation enable service ecosystem actors to integrate resources in multiple dynamic interactions to co-create value outcomes that help with population well-being and ecosystem viability.
37	Reflective technology assimilation: facilitating electronic health record assimilation in small physician practices	Baird et al [45], 2017	30	Reflective technology assimilation enables ongoing technology assimilation, such as electronic health records, by facilitating deeper learning and reflection within small organizations. Reflective action research with facilitative involvement of physicians is an efficient way to help actors create their own solution with the help of researchers.

^a^Last updated on February 26, 2024.

The keyword analysis (Table 10) is in accord with the content analysis. Apart from *EHR*, *telemedicine*, *ehealth*, *digital health*, and *telehealth*, which were part of the inclusion criteria, many related keywords appeared in the top 10, such as *HIT*, *management* (including *management*, *project management*, *network management*, *value management*, and *growth management*), *organization* (including *organization*, *organization theory*, *organizational change*, *organizational factors*, and *healthcare organization*), *data* (including *data*, *data justice*, *data interaction* and *data capitalism*), *study* (including *longitudinal study* and *exploratory study*), *adoption* (including *adoption*, *adoption model*, *adoption fit*, and *adoption intention*), *research* (including *empirical research*, *action research*, *qualitative research*, *service research*, *qualitative research* and *intervention research*), *process*, *ecosystem* (including *ecosystem analysis*, *platform ecosystem*, and *healthcare ecosystem*), *marketing*, and *strategy* (including *hospital strategy*, *strategic alignment*, and *growth strategy*).

**Table 10:** Top 10 grouped keywords of cluster 3.

Grouped keywords	Count
ehr	84
hit	37
telemedicine	35
management	31
health	27
organization	24
ehealth	19
data	15
study	13
digitalhealth	12

Taking into account the most-cited papers from this decade, we can state that transforming the health system through digitalization of the system itself or the technologies being used in patients’ care is a major challenge for academics and managers. The global business model for telehealth or digital health is filled with paradoxes, such as inefficient implementations of digital health helping patients gain control over their health. Moreover, the sector is seen as very subject to change, which makes it risky for policy makers and investors. There is also strong inertia from both patients and health professionals, as well as a lack of funding, public policies, and supportive frameworks to enhance the implementation of these telehealth technologies. Furthermore, research designs and methodologies are inappropriate for standardizing studies while including patients [46]. To address these major challenges, the ecosystem approach or the platform approach seem to be more holistic and comprehensive when looking at the health care system from an organization perspective or a structural point of view, highlighting the need for stakeholder collaboration to face complex issues at different levels [43,44].

### Cluster 4: Implementation Challenges and Ethical Considerations

The fourth cluster, represented in purple in Figure 3, had 26 nodes (5.6% of the total number of nodes) which made it the smallest cluster. With a Q3 total citation count of 13.8, more than 75% of the papers were less cited than the 50% most-cited papers of clusters 2 and 3. With a maximum total citation count of 58, an average total citation count of 9.8, and a median total citation count of 6.0, this cluster also contained some of the least-cited papers, with very low dispersion.

In terms of degree centrality, with an average degree of 6.0 and a maximum degree of 15, this was the least-connected cluster among its own nodes. It was also the least-dispersed cluster in terms of degree centrality. Again, more than 75% of these papers had a lower degree than 50% of the papers from cluster 1.

Through content analysis, we were able to label cluster 4 “implementation challenges and ethical considerations.” It examines the secondary use of EHR data and emphasizes responsible design in virtual reality rehabilitation programs. The cluster explores the role of digital health tools in empowering specific communities, such as Indigenous Australian women, and assesses the limitations and potential of mHealth technologies in health care ecosystems. Additionally, it investigates the practical aspects of technology implementation, including image quality in telehealth and the effectiveness of information tools for care coordination during patient handovers. The research papers are strongly connected with behavior change and patient empowerment through EMRs, self-tracking devices, and new uses (Table 11).

**Table 11:** Top 3 papers according to their degree centrality in Cluster 4.

Degree centrality	Title	Authors Year	Total citations^a^	Principal findings
15	Information quality life cycle in secondary use of EHR data	Hausvik et al [47], 2021	8	The information quality life cycle in the secondary use of electronic health record data can be divided in 3 processes: information generation (data extraction, data organization, and data presentation), communication, and use. Communication quality influences the actionability of the information for application and enactment.
15	Leading change: introducing an electronic medical record system to a paramedic service	Baird and Boak [48], 2016	1	Adoption of electronic medical records in a paramedic service is primarily influenced by perceived ease of use and the user interface. Barriers to acceptance are removed by introducing flexibility of use.
14	The expected and perceived well-being effects of short-term self-tracking technology use	Kari et al [49], 2017	16	Living with a self-tracking technology can have a negative influence on the daily life of the user and a negative effect on perceived well-being. Receiving positive feedback can be expected to increase well-being, while negative feedback can be expected to not have a significant impact on well-being. The perceived effect on well-being of technology is small, which is attributable to the activity tracking itself. An increase in psychological well-being helped users to continue using self-tracking devices.

^a^Last updated on February 26, 2024.

The keyword analysis (Table 12) helped to complete the content analysis. Apart from *EHR*, *mhealth*, *digital health*, *ehealth*, and *telehealth*, which were part of the inclusion criteria, keywords that appeared in the top 10 included *technology*, *apps* (including *apps*, *mobile apps*, and *health apps*), *tracking* (including *tracking*, *trackers*, *self tracking*, and *activity tracking*), *design* (including *responsible design*, *value sensitive design*, *codesign*, *design approach*, and *design science*), *data* (including *data*, *data justice*, *data interaction*, and *data capitalism*), *ethics* (including *ethics* and *guidance ethics*) and *virtual reality*.

**Table 12:** Top 10 grouped keywords in cluster 4.

Grouped keywords	Count
technology	8
apps	7
ehr	5
tracking	5
design	5
mhealth	5
health	4
digitalhealth	4
data	4
ethics	3
developingcountry	3

Taking into account the most cited papers from this decade, we can state that awareness of patients’ empowerment and ethical issues is rising more and more, and these issues need to be addressed properly. Digital technologies are empowerment tools because they allow patients to actively take control of their health. Empowerment through self-tracking devices or trackers can emphasize patient autonomy and informed decision-making while improving patients’ digital literacy [49,50]. However, mHealth or digital technologies should be considered as digital empowerment companions rather than sufficient by themselves as empowerment tools, since conceptual and ethical issues can arise and lead to issues related to medical paternalism for patients [51]. The upcoming challenge of ethical empowerment requires careful thought on ethical considerations related to privacy, consent, and informed decision-making to ensure a transition from patiency to agency for all groups of patients.

## Discussion

### Principal Findings

This study used a systematic bibliometric analysis of academic papers in the field of management centered on digital health using both performance analysis and co-citation analysis. Using modularity classes, we identified clusters of papers across all journals in the field. With an analysis of the most representative papers through their degree centrality, we were able to explain these clusters. Lastly, through an analysis of recent highly cited papers from each cluster, we identified several challenges in the literature related to digital health. We made 2 principal findings: we found 4 digital health topic clusters in management science, and we gained an understanding of their significance, the stakes, and challenges that may lead to new research questions (Table 13).

**Table 13:** Identified challenges across business and management literature about digital health.

Theme	Challenges	Potential research questions
User adoption and engagement in mHealth	Digital literacy, especially in low-income countries, affects the adoption of health technologies to improve patient empowerment and reduce social and digital inequalities	How can digital literacy be improved in low-income countries? What is the effect of trust on mHealth adoption? Can digital literacy reduce social and digital inequalities?
Adoption and trust in mHealth services	Personalized digital health depending on the population (ie, demographics or country) Factors in acceptance of the Internet of Things, telemedicine, and other digital health technologies	How can the patient experience be personalized based on user profiles? How can personalizing the patient experience aid in technology adoption?
Digital transformation in health care	Collaboration among actors to face multilevel issues in digital health through platforms, ecosystems, or other types of interorganizational collaboration structure Public policies to fully integrate digital health as an essential component of tomorrow’s health care system	How can policy makers mitigate risks associated with the rapid changes and uncertainties in the digital health sector? How can digital health research methodologies be standardized to ensure the patient-centeredness of studies and cross-study comparability? How can the ecosystem or platform approach be operationalized to foster collaboration among stakeholders in addressing digital health challenges?
Implementation challenges and ethical considerations	Ethical empowerment through privacy, consent, and an informed decision-making process	What are the ethical concerns in using digital health technologies as empowerment tools, particularly regarding privacy, consent, and informed decision-making? How can digital health tools be designed to support patient autonomy without reinforcing medical paternalism? Which frameworks or guidelines are needed to facilitate a transition from patient dependency to active agency in digital health contexts?

The most interconnected thematic area, user adoption and engagement in mHealth, was not the most globally cited. Instead, the most globally cited theme was adoption and trust in mHealth services, which explores related topics but introduces distinct challenges. Interestingly, research on user adoption has been particularly significant in low-income countries, such as Bangladesh [52-55], despite receiving fewer citations overall. Insights emerge by foregrounding digital literacy and developing dedicated tools to measure it [36]. Methodologically, models such as the UTAUT [56], TAM, or other behavioral models—widely applied in e-banking adoption, vehicle use, and social media use—have become increasingly popular in digital health adoption research [56-60].

The largest thematic area was digital transformation in health care, which comprised 47.6% of all papers in our analysis, making it the most researched field. Unsurprisingly, the widely studied theme of digitalization—explored in various fields such as construction [61], agriculture [62], industrial development [63], and even finance [64]—has also extended into health sectors such as pharmacy, hospitals, health data, and health records. A more holistic or ecosystem-oriented view of digital health has also emerged, highlighting key components of the digital health industry, including telemedicine, public health surveillance, self-tracking, medical imaging, EHRs, and privacy [5]. Public policy strategies, stakeholder collaboration, and innovation management are increasingly being recognized as critical challenges in digital health management.

Finally, the smallest thematic area was implementation challenges and ethical considerations, which was mainly composed of very recent research in the field, with over half of the papers published since 2020. These papers showed emerging challenges, such as ethical empowerment through privacy, consent, and informed decision-making [49,51]. This area deserves focused attention from researchers, policy makers, and health care managers, as it could drive key ethical practices in health technology implementation.

Additionally, our analysis sheds light on digital health business, accounting, and management papers in the secondary results of our performance analysis. Medicine and computer sciences were the two main secondary fields of study for management science papers on digital health. The main sources were *Health Care Management Review*, the *Journal Of Healthcare Management*, and the *Journal Of Commercial Biotechnology*. The papers came from various countries, mostly the United States, with a small number of papers from South America and Africa. This could lead to severe misunderstandings of local challenges to digital literacy related to trust, privacy, and perceived efficiency.

Finally, the analysis highlighted the usefulness of our methodology, which was based on selecting keywords when different concepts overlapped, meaning that the ability to choose the best keywords was not guaranteed. By comparing similar keywords in various searches, we were able to identify keywords that best represented the academic ecosystem surrounding business, accounting, and management in digital health, and we were able to execute a keyword-only search that avoided off-topic papers.

### Comparison With Prior Work

Our study diverges from previous research in several significant ways. First, unlike prior analyses that encompassed a broad spectrum of digital health literature [6], we narrowed our search specifically to the field of management science in digital health. This targeted approach allowed for a more in-depth exploration of key themes and main challenges within this specific domain that excluded many highly specific review studies about technology adoption or use [65-67]. Additionally, we used cluster analysis, leveraging network methodologies, rather than relying solely on traditional bibliometric indicators and performance analysis, such as citation counts or PageRank ranking. This nuanced approach enabled us to uncover intricate relationships and patterns among research papers within each cluster: user adoption and engagement in mHealth, adoption and trust in mHealth services, digital transformation in health care, and implementation challenges and ethical considerations. Moreover, we have provided detailed interpretations for each cluster, offering insights into the thematic content and significance of the included papers as well as upcoming challenges. In addition, our analysis of the most significant papers was conducted cluster-wise rather than globally, providing a more granular understanding of influential papers within each cluster. Furthermore, our keyword analysis explored regrouping related keywords and was performed at the cluster level, again offering a comprehensive perspective on the thematic landscape within each cluster. Importantly, we have provided statistical measures such as dispersion, average total citations, and average degree centrality, enhancing the robustness of our findings and allowing researchers to gain deeper insights into the positions within specific clusters. This approach not only fostered a better understanding of research communities but also offered valuable insights into areas of high research activity within the domain of management science in digital health.

### Limitations

First, our study focused exclusively on English-language research papers available on Scopus, a widely recognized citation database that is considered more complete in this field than other databases [20]. A worldwide analysis would include papers from other databases, such as PubMed or Web of Science, even though there would be a substantial overlap [68]. Including non–English-language research papers, especially those written in Chinese, could be beneficial.

Second, due to the vast number of papers included in our analysis, it was not feasible to comprehensively review each paper one by one, so we had to select the papers that were the most representative of each cluster. Using artificial intelligence to summarize the papers could have helped us include more of them in the analysis without having to select representative papers.

Third, there is no universally agreed-upon method to rank papers and identify the most representative papers within a cluster; various tools are used according to the goal of the study [17]. As a result, we explored various metrics, such as co-citation, PageRank, total citations, and degree centrality in the network to assess the significance of papers.

Fourth, the clusters varied significantly in size, making them less comparable, and certain papers were on the border of multiple clusters depending on the cluster algorithm used or the parameters in the Louvain algorithm. To partially overcome this limitation, we removed papers that were in very small clusters and read every paper located on a border to understand how to link the clusters. Since the Louvain algorithm is partially random, there was no way to ensure that a new iteration did not result in slight differences.

Last, it is worth noting that papers from low-income countries were relatively underrepresented in our dataset. Further analysis is needed of papers from these underrepresented countries to understand whether there are differences in the main interests in the digital health management literature and whether digital health management is not a primary focus because of other sociopolitical factors.

### Future Directions

Research in digital health in business management is a very heterogeneous field that one might formalize by adding subgroups such as user adoption and engagement in mHealth, adoption and trust in mHealth services, digital transformation in health care, and implementation challenges and ethical considerations. This last research domain is also the most recent, and it could benefit from more research to determine whether ethical considerations are a motor of implementation of digital health in services, ecosystems, and societies to help cope with important challenges.

We also consider that a more longitudinal analysis could help to understand the dynamics of how clusters emerge and allow anticipating subjects that could benefit from more research, such as ethics, empowerment, and literacy in low-income countries. Such a study could also benefit from including papers in the field of medicine as well as management science to be more complete.

## Appendix

Multimedia Appendix 1 Keywords selection methodology with comparison of keywords for 2 requests.

Multimedia Appendix 2 All nodes data from clusters 1 to 4.

Multimedia Appendix 3 Complete secondary subject area of publication analysis.

Multimedia Appendix 4 Complete source of publication analysis.

Multimedia Appendix 5 Complete list of the 160 most represented keywords for the keyword analysis.

Multimedia Appendix 6 All grouping of keywords for the keyword analysis.

Multimedia Appendix 7 Complete country and zone of publication analysis.

## Abbreviations

EHR: electronic health record
EIQR: extended IQR
EMR: electronic medical record
HIT: health IT
mHealth: mobile health
Q1: first quartile
Q3: third quartile
TAM: technology acceptance model
UTAUT: unified theory of acceptance and use of technology

## References

[1] (2020). What is digital health?. US Food and Drug Administration.

[2] Fatehi F, Samadbeik M, Kazemi A (2020). What is digital health? Review of definitions. Stud Health Technol Inform.

[3] Minvielle E (2015). Santé numérique: enquête sur une révolution annoncée. Le Libellio d'AEGIS.

[4] Moerenhout T, Devisch I, Cornelis GC (2018). E-health beyond technology: analyzing the paradigm shift that lies beneath. Med Health Care Philos.

[5] Iyawa GE, Herselman M, Botha A (2016). Digital health innovation ecosystems: from systematic literature review to conceptual framework. Procedia Comp Sci.

[6] Ahmadvand A, Kavanagh D, Clark M, Drennan J, Nissen L (2019). Trends and visibility of "digital health" as a keyword in articles by JMIR publications in the new millennium: bibliographic-bibliometric analysis. J Med Internet Res.

[7] Eysenbach G (2001). What is e-health?. J Med Internet Res.

[8] Peng C, He M, Cutrona SL, Kiefe CI, Liu F, Wang Z (2020). Theme trends and knowledge structure on mobile health apps: bibliometric analysis. JMIR Mhealth Uhealth.

[9] Morley J, Machado CC, Burr C, Cowls J, Joshi I, Taddeo M, Floridi L (2020). The ethics of AI in health care: A mapping review. Soc Sci Med.

[10] Tortorella GL, Fogliatto FS, Saurin TA, Tonetto LM, McFarlane D (2022). Contributions of Healthcare 4.0 digital applications to the resilience of healthcare organizations during the COVID-19 outbreak. Technovation.

[11] Soto-Acosta P (2020). COVID-19 pandemic: shifting digital transformation to a high-speed gear. Inf Syst Manag.

[12] Klein VB, Todesco JL (2021). COVID-19 crisis and SMEs responses: the role of digital transformation. Knowl Process Manag.

[13] Ciasullo MV, Orciuoli F, Douglas A, Palumbo R (2022). Putting Health 4.0 at the service of society 5.0: exploratory insights from a pilot study. Soc Econ Plann Sci.

[14] Grant MJ, Booth A (2009). A typology of reviews: an analysis of 14 review types and associated methodologies. Health Info Libr J.

[15] Snider C, Gopsill JA, Jones SL, Emanuel L, Hicks BJ (2019). Engineering project health management: a computational approach for project management support through analytics of digital engineering activity. IEEE Trans Eng Manage.

[16] Schwarzmüller T, Brosi P, Duman D, Welpe IM (2018). How does the digital transformation affect organizations? key themes of change in work design and leadership. Manag Revue.

[17] Donthu N, Kumar S, Mukherjee D, Pandey N, Lim WM (2021). How to conduct a bibliometric analysis: An overview and guidelines. J Bus Res.

[18] Henderson J (2005). Google Scholar: a source for clinicians?. CMAJ.

[19] Falagas ME, Pitsouni EI, Malietzis GA, Pappas G (2008). Comparison of PubMed, Scopus, Web of Science, and Google Scholar: strengths and weaknesses. FASEB J.

[20] Mongeon P, Paul-Hus A (2015). The journal coverage of Web of Science and Scopus: a comparative analysis. Scientometrics.

[21] Bankar R (2019). Bibexcel tutorial. ResearchGate.

[22] Ding Y, Yan E, Frazho A, Caverlee J (2009). PageRank for ranking authors in co‐citation networks. J Am Soc Inf Sci.

[23] Blondel VD, Guillaume J, Lambiotte R, Lefebvre E (2008). Fast unfolding of communities in large networks. J Stat Mech.

[24] Radicchi F, Castellano C, Cecconi F, Loreto V, Parisi D (2004). Defining and identifying communities in networks. Proc Natl Acad Sci U S A.

[25] Sharplin A, Mabry R (1985). The relative importance of journals used in management research: an alternative ranking. Hum Relat.

[26] MacRoberts MH, MacRoberts BR (1989). Problems of citation analysis: a critical review. J Am Soc Inf Sci.

[27] Health IT Legislation. HealthIT.gov.

[28] (2010). A digital agenda for Europe. EU4Digital.

[29] Global observatory for eHealth. World Health Organization.

[30] Directory of eHealth policies survey. World Health Organization.

[31] Alam MZ, Alam MMD, Uddin MA, Mohd Noor NA (2020). Do mobile health (mHealth) services ensure the quality of health life? An integrated approach from a developing country context. J Mark Commun.

[32] Sampat B, Prabhakar B, Yajnik N, Sharma A (2020). Adoption of mobile fitness and dietary apps in India: an empirical study. Int J Bus Inf Syst.

[33] Alam MZ, Hoque MR, Hu W, Barua Z (2020). Factors influencing the adoption of mHealth services in a developing country: A patient-centric study. Int J Inf Manag.

[34] Hamari J, Koivisto J (2015). Why do people use gamification services?. Int J Inf Manag.

[35] Zhao Y, Ni Q, Zhou R (2018). What factors influence the mobile health service adoption? A meta-analysis and the moderating role of age. Int J Inf Manag.

[36] Karnoe KA, Kayser L (2015). How is eHealth literacy measured and what do the measurements tell us? A systematic review. Know Manag E Learn.

[37] Bol N, Helberger N, Weert JCM (2018). Differences in mobile health app use: A source of new digital inequalities?. Info Soc.

[38] Xiaofei Z, Guo X, Ho SY, Lai K, Vogel D (2021). Effects of emotional attachment on mobile health-monitoring service usage: An affect transfer perspective. Info Manag.

[39] Rajak M, Shaw K (2021). An extension of technology acceptance model for mHealth user adoption. Tech Soc.

[40] Arfi WB, Nasr IB, Kondrateva G, Hikkerova L (2021). The role of trust in intention to use the IoT in eHealth: Application of the modified UTAUT in a consumer context. Technol Forecast Soc Change.

[41] Guo X, Zhang X, Sun Y (2016). The privacy–personalization paradox in mHealth services acceptance of different age groups. Electron Commer Res Appl.

[42] Kamal SA, Shafiq M, Kakria P (2020). Investigating acceptance of telemedicine services through an extended technology acceptance model (TAM). Tech Soc.

[43] Hermes S, Riasanow T, Clemons EK, Böhm M, Krcmar H (2020). The digital transformation of the healthcare industry: exploring the rise of emerging platform ecosystems and their influence on the role of patients. Bus Res.

[44] Beirão G, Patrício L, Fisk RP (2017). Value cocreation in service ecosystems: investigating health care at the micro, meso, and macro levels. J Serv Manag.

[45] Baird A, Davidson E, Mathiassen L (2017). Reflective technology assimilation: facilitating electronic health record assimilation in small physician practices. J Manag Inf Syst.

[46] Standing C, Standing S, McDermott M, Gururajan R, Kiani Mavi R (2016). The paradoxes of telehealth: a review of the literature 2000–2015. Syst Res.

[47] Hausvik GI, Thapa D, Munkvold BE (2021). Information quality life cycle in secondary use of EHR data. Int J Inf Manag.

[48] Baird S, Boak G (2016). Leading change: introducing an electronic medical record system to a paramedic service. Lead Health Serv.

[49] Kari T, Moilanen P, Makkonen M, Koivunen S, Frank L (2017). The expected and perceived well-being effects of short-term self-tracking technology use. Int J Netw Virt Organ.

[50] Maxwell H, O’Shea M, Stronach M, Pearce S (2019). Empowerment through digital health trackers: an exploration of Indigenous Australian women and physical activity in leisure settings. Ann Leis Res.

[51] Morley J, Floridi L (2020). The limits of empowerment: how to reframe the role of mHealth tools in the healthcare ecosystem. Sci Eng Ethics.

[52] Alam MZ, Hu W, Hoque MR, Kaium MA (2020). Adoption intention and usage behavior of mHealth services in Bangladesh and China. Int J Pharm Healthc Mark.

[53] Hossain MA (2016). Assessing m-Health success in Bangladesh. J Enterp Inf Manag.

[54] Zobair KM, Sanzogni L, Sandhu K (2020). Telemedicine healthcare service adoption barriers in rural Bangladesh. Australas J Inf Syst.

[55] Hossain A, Quaresma R, Rahman H (2019). Investigating factors influencing the physicians’ adoption of electronic health record (EHR) in healthcare system of Bangladesh: An empirical study. Int J Inf Manag.

[56] Williams MD, Rana NP, Dwivedi YK (2015). The unified theory of acceptance and use of technology (UTAUT): a literature review. J Enterp Inf Manag.

[57] Barua Z, Barua A (2021). Acceptance and usage of mHealth technologies amid COVID-19 pandemic in a developing country: the UTAUT combined with situational constraint and health consciousness. J Enabl Technol.

[58] Dash A, Sahoo AK (2022). Exploring patient's intention towards e-health consultation using an extended UTAUT model. J Enabl Technol.

[59] Duarte P, Pinho JC (2019). A mixed methods UTAUT2-based approach to assess mobile health adoption. J Bus Res.

[60] Kohnke A, Cole ML, Bush R (2014). Incorporating UTAUT Predictors for Understanding Home Care Patients' and Clinician's Acceptance of Healthcare Telemedicine Equipment. J Technol Manag Innov.

[61] Akinlolu M, Haupt TC, Edwards DJ, Simpeh F (2020). A bibliometric review of the status and emerging research trends in construction safety management technologies. Int J Constr Manag.

[62] Klerkx L, Jakku E, Labarthe P (2022). A review of social science on digital agriculture, smart farming and agriculture 4.0: New contributions and a future research agenda. NJAS Wageningen J Life Sci.

[63] Beier G, Ullrich A, Niehoff S, Reißig M, Habich M (2020). Industry 4.0: How it is defined from a sociotechnical perspective and how much sustainability it includes – A literature review. J Clean Prod.

[64] Bollaert H, Lopez-de-Silanes F, Schwienbacher A (2021). Fintech and access to finance. J Corp Finance.

[65] Singh H, Tang T, Steele Gray C, Kokorelias K, Thombs R, Plett D, Heffernan M, Jarach CM, Armas A, Law S, Cunningham HV, Nie JX, Ellen ME, Thavorn K, Nelson ML (2022). Recommendations for the design and delivery of transitions-focused digital health interventions: rapid review. JMIR Aging.

[66] Wegener EK, Bergschöld Jenny M, Whitmore C, Winters M, Kayser L (2023). Involving older people with frailty or impairment in the design process of digital health technologies to enable aging in place: scoping review. JMIR Hum Factors.

[67] Gabarron E, Larbi D, Rivera-Romero O, Denecke K (2024). Human factors in AI-driven digital solutions for increasing physical activity: scoping review. JMIR Hum Factors.

[68] Kumpulainen M, Seppänen M (2022). Combining Web of Science and Scopus datasets in citation-based literature study. Scientometrics.

